# Intestinal proline is a potential anti-allergy factor for allergy diagnosis and therapy

**DOI:** 10.3389/fnut.2022.1036536

**Published:** 2022-11-30

**Authors:** Jinya Ma, Chong Wang, Fangting Wang, Yong Zhang, Yinghua Liu, Jiachao Zhang, Zhongshan Gao, Yi Zhang, Hujun Xie, Yanbo Wang, Linglin Fu

**Affiliations:** ^1^Food Safety Key Laboratory of Zhejiang Province, School of Food Science and Biotechnology, Zhejiang Gongshang University, Hangzhou, China; ^2^Department of Nutrition, The First Medical Center of People’s Liberation Army (PLA) General Hospital, Beijing, China; ^3^Key Laboratory of Food Nutrition and Functional Food of Hainan Province, College of Food Science and Engineering, Hainan University, Haikou, China; ^4^Allergy Research Center, Zhejiang University, Hangzhou, China; ^5^College of Food Science, Fujian Agriculture and Forestry University, Fuzhou, China

**Keywords:** proline, allergy, biomarker, IgE, cohort study

## Abstract

Allergy has become a public health problem worldwide, but effective diagnostic and therapeutic approaches are limited currently. Amino acids are essential macronutrients that potentially participated in the allergy process. This work aimed to investigate whether amino acids can be applied as a mediator for allergy diagnosis and therapy. Two cohort studies were performed to investigate the correlation between fecal amino acids and allergy responses, and a spleen cell model was used to validate the role of amino acids in regulating allergy. In a cohort study with 193 volunteers, fecal proline was found to be negatively correlated with serum IgE, and detailed data analysis revealed that people with high-IgE-mediated allergy had decreased odds of high intestinal proline. In another cohort study with distinct allergic and non-allergic individuals, proline concentration was significantly lower in the allergic group. Daily diet and metagenomics analysis showed that the proline intake and microbiota amino acid metabolism were not significantly different, implying that the body’s proline metabolism might be different between allergic and non-allergic individuals. Furthermore, the spleen cell model demonstrated that proline specifically targeted Th2 and Treg activity. Overall, this work revealed a tight correlation between gut proline and serum IgE, indicating proline as a promising biomarker and a potential therapeutic method for allergic diseases.

## Introduction

Allergy has become a public health problem with increasing prevalence worldwide, which afflicts 10–40% population in different countries worldwide, and the prevalence keeps increasing rapidly (1). Although a lot of effort has been put into controlling allergic diseases, effective diagnostic, and therapeutic approaches are limited currently (2–4).

Diagnosing allergic diseases is the basis for controlling them. Skin test (such as skin prick test) and oral challenge test (for food allergy) are commonly used *in vivo* diagnostic methods for allergy, but these approaches may show false positive outcomes and have potential risks of systemic reactions (5). In addition, professional and experienced operators are required to perform the tests, which limits the application situations. On the other hand, *in vitro* tests based on biomarkers are more convenient and safer diagnostic methods and are thus more suitable for some application situations such as large-scale screening. IgE (both non-specific and allergen-specific) is the most commonly used biomarker for allergy tests, while histamine and eosinophilia were also capable to indicate allergic responses in some situations (5, 6). However, measuring these biomarkers requires blood samples, and the results are less reliable than those from *in vivo* tests in most cases, and more reliable, convenient, and non-destructive biomarkers that are suitable for large-scale screening are currently absent.

Several therapeutic methods have been developed for allergies. For example, antihistamines and anti-IgE monoclonal antibodies are efficient treatment methods for allergic diseases, but the duration is relatively short, and the side effects cannot be ignored (7). On the other hand, allergen immunotherapy is a long-term effective method, but the allergen-specificity avoids broad-spectrum anti-allergic effects, and the potential risks also limited the applications (8).

Therefore, novel diagnostic and therapeutic methods for allergies are urgently needed. The elucidation of *in vivo* targets will facilitate the development of novel control approaches for allergic diseases. Amino acids are essential macronutrients for humans, it has been reported that particular amino acids can regulate the immune system (9), and some amino acids in the gut were correlated with allergic responses (10). These finds suggested amino acids to be anti-allergic candidates and potential allergy biomarkers, but knowledge of the direct correlation between allergy and amino acids is limited.

Taking together, this work aims to reveal novel fecal biomarkers for allergy. The amino acid content in the feces from two cohort studies was assessed, and the involvement in allergic responses was analyzed. Finally, proline was found to be tightly correlated with allergic responses and total IgE concentration, implying it to be a potential biomarker for controlling allergy.

## Materials and methods

### Cohort study

#### Cohort study A

A total of 193 adults were recruited in this experiment. All participants were between 18 and 24 years old. The participants answered a questionnaire that addressed their physiological characteristics, healthy condition (including allergy), and typical dietary intake in the 2 weeks before feces sampling.

#### Cohort study B

A total of 19 adults were recruited in this experiment, including 11 non-allergic volunteers and eight allergic volunteers. All participants were between 18 and 25 years old. The non-allergic volunteers did not have any self-reported allergic diseases and did not have any significant allergy-like symptoms in the past 2 years, and the allergic volunteers were diagnosed by the hospital and would show significant allergic symptoms when in contact with particular allergens. None of the subjects had a gastric, gut, or metabolic disease, and had not taken antibiotics in the 2 months before the study. The participants answered a questionnaire that addressed their physiological characteristics, healthy condition, and typical dietary intake in the 2 weeks before feces sampling.

#### Sample collection

Human fresh fecal material was collected with a disposable sampling spoon into a 50 ml sterile enzyme-free centrifuge tube. Human serum samples were collected in the medical office of Zhejiang Gongshang University. After placing the whole blood in the centrifuge for 30 min at 4000 rpm, the upper serum layer was saved and placed in a 2 ml sterile enzyme-free centrifuge tube. The samples were aliquoted and stored at –80°C for subsequent experiments.

### Determination of amino acid content

The concentrations of amino acids (SER, GLY, HIS, ARG, THR, ALA, PRO, TYR, VAL, MET, CYS, ILE, LEU, PHE, LYS, ASP + ASN, and GLU + GLN) in feces were determined with a Hitachi L-8900 automatic amino acid analyzer (Hitachi, Tokyo, Japan). The feces samples were dried in an oven at 60°C and crushed through an 80-mesh sieve. 100 mg sample was moved to a hydrolysis tube, and 4 ml 1:1 analytical pure hydrochloric acid (about 6 mol/L) was added. The hydrolysis tube was then blown with nitrogen for 15 min and sealed immediately. The sealed sample was heated in an oven at 110°C for 24 h to hydrolyze. Then the sample was filtrated into a 100 ml volumetric flask and diluted to 100 ml by water. 2 ml sample was put on the nitrogen-blowing instrument to dry and reconstitute in 2 ml 0.02 mol/L HCl. Then the sample was filtrated by a 0.22 μm organic filter membrane, and the amino acid content was determined.

### Enzyme-linked immunosorbent

IgG1, IgG2, and IgE in serum samples were measured with human immunoglobulin enzyme-linked immunosorbent (ELISA) kits (Cat# 88-50560-22, 88-50570-22, and 88-50610-22, respectively, Thermo Fisher Scientific, Waltham, USA) following the instructions. IgA and histamine was measured with secretory immunoglobulin A ELISA kit and histamine assay kit (Cat# H108-2 and H171, Nanjing Jiancheng Bioengineering Institute, Nanjing, China) following the manufacturer’s instruction. The absorbance was determined with a VersaMax microplate reader (Molecular Devices, San Jose, USA) at 450 nm, and the concentration was quantified based on the standard curve according to the instruction of the kits.

### Metagenomic analysis

Genomic DNA was extracted from fecal samples using the QIAGEN DNA Stool Mini-Kit (QIAGEN, Hilden, Germany) following the manufacturer’s instructions. One microgram DNA per sample was used as the input material for DNA sample preparations. Sequencing libraries were generated using NEB Next^®^ Ultra™ DNA Library Prep Kit for Illumina (NEB, Ipswich, USA) following the manufacturer’s recommendations and index codes were added to attribute sequences to each sample. Briefly, the DNA sample was fragmented by sonication to a size of 350 bp, then DNA fragments were end-polished, A-tailed, and ligated with the full-length adaptor for Illumina sequencing with further PCR amplification. At last, PCR products were purified (AMPure XP system) and libraries were analyzed for size distribution by Agilent 2100 Bioanalyzer and quantified using real-time PCR. The clustering of the index-coded samples was performed on a cBot Cluster Generation System according to the manufacturer’s instructions. After cluster generation, the library preparations were sequenced on an Illumina HiSeq platform and paired-end reads were generated.

The shotgun reads were assembled into contigs and scaffolds using IDBA-UD (11), and then the contigs were used to predict the functional genes with MetaGeneMark (12). Finally, a non-redundant gene catalog was constructed using CD-HIT (13), and the abundances of genes were determined by aligning the reads to the gene catalog using Bowtie 2 (14).

The microbial taxonomic profile was constructed by the software MetaPhlAn2 (15). For metagenomic species analysis, the software MetaBAT (16) was applied, and the co-abundance principle and canopy clustering algorithm were performed to generate MAGs (metagenomic assembled genomes) by binning shotgun reads. After reassembling, the MAGs were assigned to a given genome when more than 80% of the subgene matched the same genome using BLASTn at a threshold of 95% identity over 90% of the gene length. If greater than 80% of the genes from a MAG had the same taxonomic level of assignment, then MGS was identified as the same microbe.

The annotated sequences were assigned to the KEGG orthologue group (KO) according to the highest score. Reporter Z-scores were calculated to reveal the differences in enriched metabolic pathways between groups, a reporter score of >2.3 (90% confidence according to the normal distribution) was used as a detection threshold to significantly differentiate between pathways (17).

### Cell model

The fresh spleen isolated from healthy mice were aseptically minced by syringe coring on a 200-screen mesh cell strainer and then treated with red blood cell lysis buffer (Sangon Biotech, Shanghai, China) to remove red blood cells. After subsequent washing by PBS, the single-cell suspension was resuspended in RPMI 1640 medium (Thermo Fisher Scientific, Waltham, USA) supplemented with 10% fetal bovine serum (Thermo Fisher, Waltham, USA), 100 U/ml penicillin, and 100 mg/ml streptomycin (Sangon Biotech, Shanghai, China) and counted. Then, the cells were cultured in 12-well plates (Costar, Corning Life Sciences, Tewksbury, USA) at a concentration of 5 × 10^6^ per well (1 ml per well) at 37°C with 5% CO_2_ for 24 h. Then 40 mM proline was added to the cells, after further culturing for 24 h, the cells were harvested, and the gene expression was determined by qPCR. The primers sequence was listed in Supplementary Table 1.

## Results and discussion

### Intestinal proline correlated with allergy

To investigate the potential correlation between amino acid metabolism and allergy, we performed a cohort study with 193 volunteers (cohort study A) (Table 1). The feces amino acid content was quantified by HPLC, and the main allergy-related indexes were quantified by ELISA. Pearson correlation analysis showed a significant negative correlation between proline and IgE was revealed (*r* = –0.151, *p* = 0.038), whereas, no significant correlation between proline and IgG1, IgG2, and IgA, or between other amino acids and immunoglobins, was obtained (Supplementary Table 2). This result indicated a specific relationship between intestinal proline and IgE-mediated allergy.

**TABLE 1:** Demographics of the volunteers.

	Cohort study A (*N* = 193)	Cohort study B	
		
		Non-allergic group (*N* = 11)	Allergic group (*N* = 8)
Age (year)^a^	20.00 ± 0.06	23.64 ± 0.15	22.50 ± 0.71
Gender (male, %)	46 (23.8%)	4 (36.4%)	1 (12.5%)
Serum IgE (ng/ml)^a^	66.27 ± 6.74	29.88 ± 5.93	61.80 ± 10.79

^a^Data are expressed as mean ± SEM.

**FIGURE 1:**
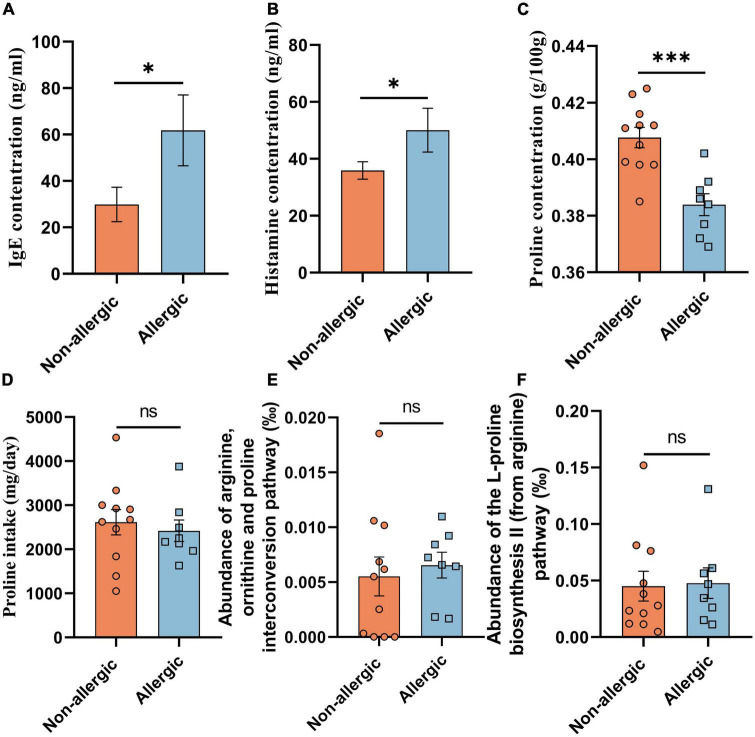
Intestinal proline correlated with allergy. The concentration of serum IgE **(A)**, serum histamine **(B)**, feces proline **(C)**, and daily diet proline intake **(D)** of the volunteers in cohort study B were determined. And the abundance of the arginine, ornithine and proline interconversion pathway **(E)** and the abundance of the L-proline biosynthesis II (from arginine) pathway **(F)** were measured based on functional KO annotation of the metagenome. Data were presented as mean ± SEM. The statistical differences were calculated by Student’s *t*-test. *p < 0.05; ***p < 0.001; ns, not significant.

We further recruited 11 non-allergic volunteers and 8 allergic volunteers in another cohort study (cohort study B) (Table 1). Serum IgE and histamine levels confirmed the significant difference between the two groups (Figures 1A,B). The feces proline was significantly lower in the allergic group, confirming that intestinal proline concentration is negatively correlated with allergy (Figure 1C). Moreover, the proline intake was estimated by dietary information (Figure 1D) and the microbiota diversity and metabolism (Supplementary Figures 1A,B and Figures 1E,F) were assessed by metagenomic sequencing, but none showed a significant difference between allergic and non-allergic groups, and only limited strains showed significantly different abundance between the two groups (Supplementary Figure 1C). Although these results cannot rule out that different dietary habits or microbiota metabolism could regulate intestinal proline, it implied that the body proline metabolism might be different between allergic and non-allergic individuals.

### Proline down-regulates IgE concentration

**FIGURE 2:**
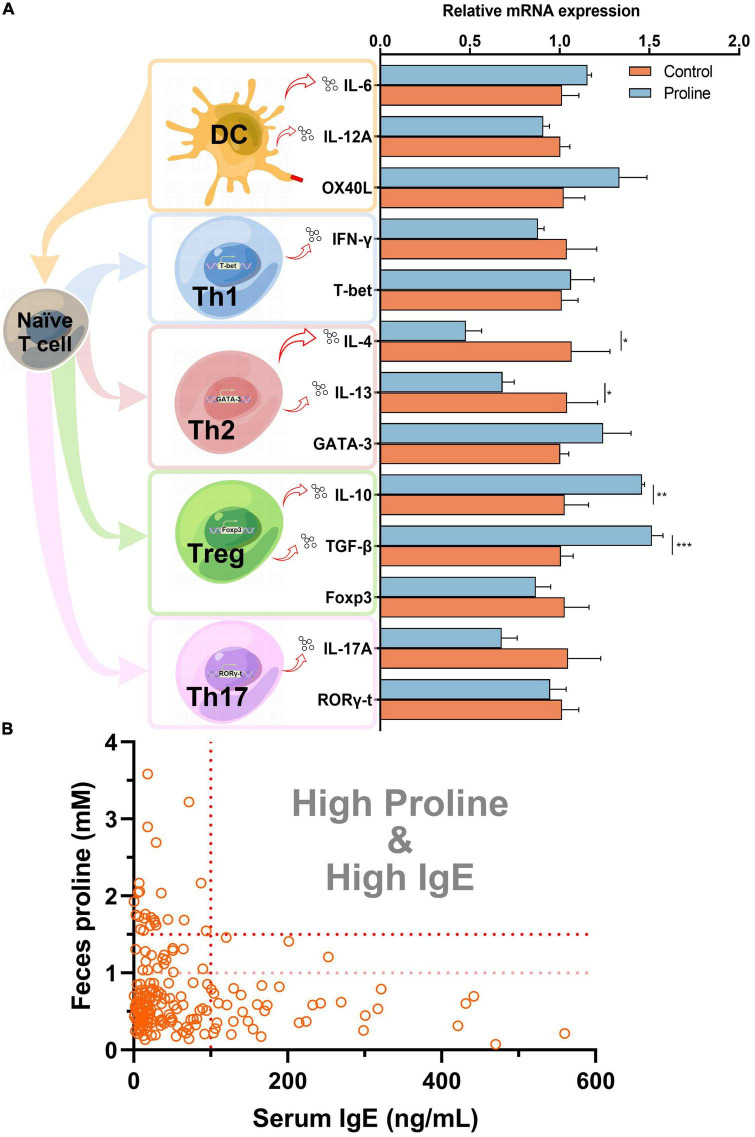
Proline down-regulates IgE concentration. **(A)** Mice spleen cells were treated by proline and the mRNA transcription of allergy-related proteins was determined by qPCR. **(B)** The concentration of feces proline and serum IgE of each individual in the cohort study. Data were presented as mean ± SEM. The statistical differences were calculated by Student’s *t*-test. *p < 0.05; **p < 0.01; ***p < 0.001; ns, not significant.

To understand how proline regulates allergic responses, a cell culture model was established by using spleen cells from healthy mice. As shown in Figure 2A, the transcription of Th2 cytokines IL-4 and IL-13 were significantly down-regulated after proline treatment, while the transcription of Treg cytokines IL-10 and TGF-β was up-regulated. Noticeably, the mRNA transcription of other proteins, including those mainly expressed by Th1 (INF-γ and T-bet), Th17 (IL-17A and ROR-γt), and DC (IL-6, IL-12A, and OX40L), were not changed, and the crucial transcription factors of Th2 (GATA-3) and Treg (Foxp3) was also unchanged. This result implied that proline specifically targeted Th2 and Treg activity. Based on this, whether the administration of proline will regulate these cells and then alleviate allergic responses was an interesting problem to be further investigated.

Finally, we analyzed the concentration of proline and IgE in each individual in cohort study A. Interestingly, for those individuals with a higher concentration of proline (>1.5 mM), their serum IgE levels were all lower than 100 ng/ml (Figure 2B), which could be regarded as total IgE negative (18). Furthermore, the result also revealed that people with high-IgE-mediated allergy had decreased odds of high intestinal proline (Table 2). Based on this, feces proline was a promising marker for allergy diagnosis, especially for large-scale screening, which can rapidly exclude a large proportion of IgE-negative individuals.

**TABLE 2:** Odds ratio analysis of high-IgE based on feces proline concentration.

	Value	95% confidence interval	
		
		Lower	Upper
Odds ratio for proline (>1 mM:<1 mM)	0.241	0.070	0.828
For cohort IgE > 100 ng/ml	0.293	0.094	0.908
For cohort IgE < 100 ng/ml	1.215	1.077	1.369
Pearson Chi-Square	5.848	*p* = 0.016*	

*p < 0.05.

In conclusion, our study revealed a tight correlation between gut proline and serum IgE, indicating proline as a promising biomarker for IgE-mediated allergy. Proline suppresses Th2 cells and activates Tregs, therefore is a potential therapeutic approach to allergic diseases. Although the detailed underlying mechanisms required further elucidation, this work is a pilot study that provided intestinal proline as a promising subject for further allergy investigations.

## Data availability statement

The data presented in the study are deposited in the NCBI repository, accession number PRJNA902178.

## Ethics statement

The studies involving human participants were reviewed and approved by Zhejiang Gongshang University Ethics Review Committee. The patients/participants provided their written informed consent to participate in this study.

## Author contributions

LF, YL, YiZ, and YW designed the research. CW, YoZ, JZ, and HX analyzed the data. JM, FW, and ZG performed the research. JM and CW wrote the manuscript. All authors contributed to the article and approved the submitted version.
