# Supplementary material for: Intestinal proline is a potential anti-allergy factor for allergy diagnosis and therapy
Source: Front Nutr. 2022 Nov 30;9:1036536. doi: 10.3389/fnut.2022.1036536 (PMC9748422; doi:10.3389/fnut.2022.1036536)
Supplement: Supplementary file 1 [file Data_Sheet_1.DOCX]

# Supplementary material

**Supplementary Table 1. Primers for qPCR**

| Genes | Forward Primer (5’-3’) | Reverse Primer (5’-3’) |
| --- | --- | --- |
| β-actin | CTACCTCATGAAGATCCTGACC | CACAGCTTCTCTTTGATGTCAC |
| OX40L | CCCTCCAATCCAAAGACTCA | ATCCTTCGACCATCGTTCAG |
| IL-6 | CCGGAGAGGAGACTTCACAG | TCCACGATTTCCCAGAGAAC |
| IL-12 | GCACTTGATAGGCAGAGGCAGATG | GCACTGGCTGTCCTGGAACTTAC |
| T-bet | TCAACCAGCACCAGACAGAG | AACATCCTGTAATGGCTTGTG |
| IFN-γ | TGGCATAGATGTGGAAGAAAAGAG | TGCAGGATTTTCATGTCACCA |
| GATA3 | CTTATCAAGCCCAAGCGAAG | CCCATTAGCGTTCCTCCTC |
| IL-4 | ACAGGAGAAGGGACGCCAT | GAAGCCCTACAGACGAGCTCA |
| IL-13 | AGACCAGACTCCCCTGTGCA | TGGGTCCTGTAGATGGCATTG |
| Foxp3 | TTTCACCTATGCCACCCTTATC | CATGCGAGTAAACCAATGGTAG |
| IL-10 | GGTTGCCAAGCCTTATCGGA | ACCTGCTCCACTGCCTTGCT |
| TGF-β | GGTTGCCAAGCCTTATCGGA | ACCTGCTCCACTGCCTTGCT |
| RORγt | AGAAAGAAAAGGGGAACTGG | CTATTGTGGCTGCTGAGTTC |
| IL-17a | AGGGAGAGCTTCATCTGTGG | AGATTCATGGACCCCAACAG |

**Supplementary Table 2. Pearson correlation analysis between serum antibody isotypes and feces amino acids** **of cohort study A**

| **Amino acid** |  | **IgE** | **Histamine** | **IgG1** | **IgG2** | **IgA** |
| --- | --- | --- | --- | --- | --- | --- |
| PRO | r | -.151* | -0.032 | -0.042 | -0.059 | -0.127 |
|  | p | 0.038 | 0.662 | 0.571 | 0.418 | 0.095 |
| ASP+ASN | r | 0.045 | 0.085 | -0.046 | 0.054 | -0.112 |
|  | p | 0.538 | 0.242 | 0.531 | 0.455 | 0.141 |
| GLU+GLN | r | 0.029 | 0.053 | -0.055 | 0.028 | -0.121 |
|  | p | 0.687 | 0.463 | 0.460 | 0.704 | 0.110 |
| SER | r | -0.018 | 0.072 | -0.095 | 0.023 | -0.101 |
|  | p | 0.807 | 0.317 | 0.199 | 0.751 | 0.185 |
| GLY | r | -0.091 | -0.032 | -0.106 | 0.021 | -0.145 |
|  | p | 0.212 | 0.657 | 0.150 | 0.771 | 0.056 |
| HIS | r | -0.034 | 0.019 | -0.127 | 0.010 | -0.125 |
|  | p | 0.645 | 0.796 | 0.086 | 0.893 | 0.099 |
| ARG | r | -0.047 | 0.020 | -0.075 | -0.013 | -0.112 |
|  | p | 0.520 | 0.785 | 0.313 | 0.862 | 0.139 |
| THR | r | -0.042 | 0.015 | -0.089 | 0.028 | -0.110 |
|  | p | 0.563 | 0.837 | 0.229 | 0.695 | 0.147 |
| ALA | r | -0.055 | -0.010 | -0.110 | 0.031 | -0.141 |
|  | p | 0.452 | 0.894 | 0.135 | 0.665 | 0.063 |
| TYR | r | -0.042 | -0.004 | -0.103 | 0.008 | -0.110 |
|  | p | 0.565 | 0.959 | 0.161 | 0.908 | 0.146 |
| VAL | r | -0.071 | -0.022 | -0.106 | -0.007 | -0.115 |
|  | p | 0.332 | 0.760 | 0.151 | 0.928 | 0.130 |
| MET | r | -0.060 | -0.012 | -0.097 | -0.053 | -0.099 |
|  | p | 0.412 | 0.866 | 0.189 | 0.467 | 0.193 |
| CYS | r | -0.067 | -0.025 | -0.109 | 0.021 | -0.119 |
|  | p | 0.358 | 0.727 | 0.141 | 0.770 | 0.117 |
| ILE | r | -0.092 | -0.043 | -0.096 | -0.046 | -0.085 |
|  | p | 0.205 | 0.552 | 0.192 | 0.522 | 0.262 |
| LED | r | -0.057 | -0.014 | -0.097 | 0.029 | -0.128 |
|  | p | 0.433 | 0.843 | 0.187 | 0.693 | 0.092 |
| PHE | r | -0.043 | -0.027 | -0.083 | 0.000 | -0.132 |
|  | p | 0.559 | 0.708 | 0.259 | 0.996 | 0.081 |
| LYS | r | -0.038 | -0.057 | -0.090 | 0.029 | -0.099 |
|  | p | 0.602 | 0.430 | 0.221 | 0.689 | 0.192 |

* p < 0.05.


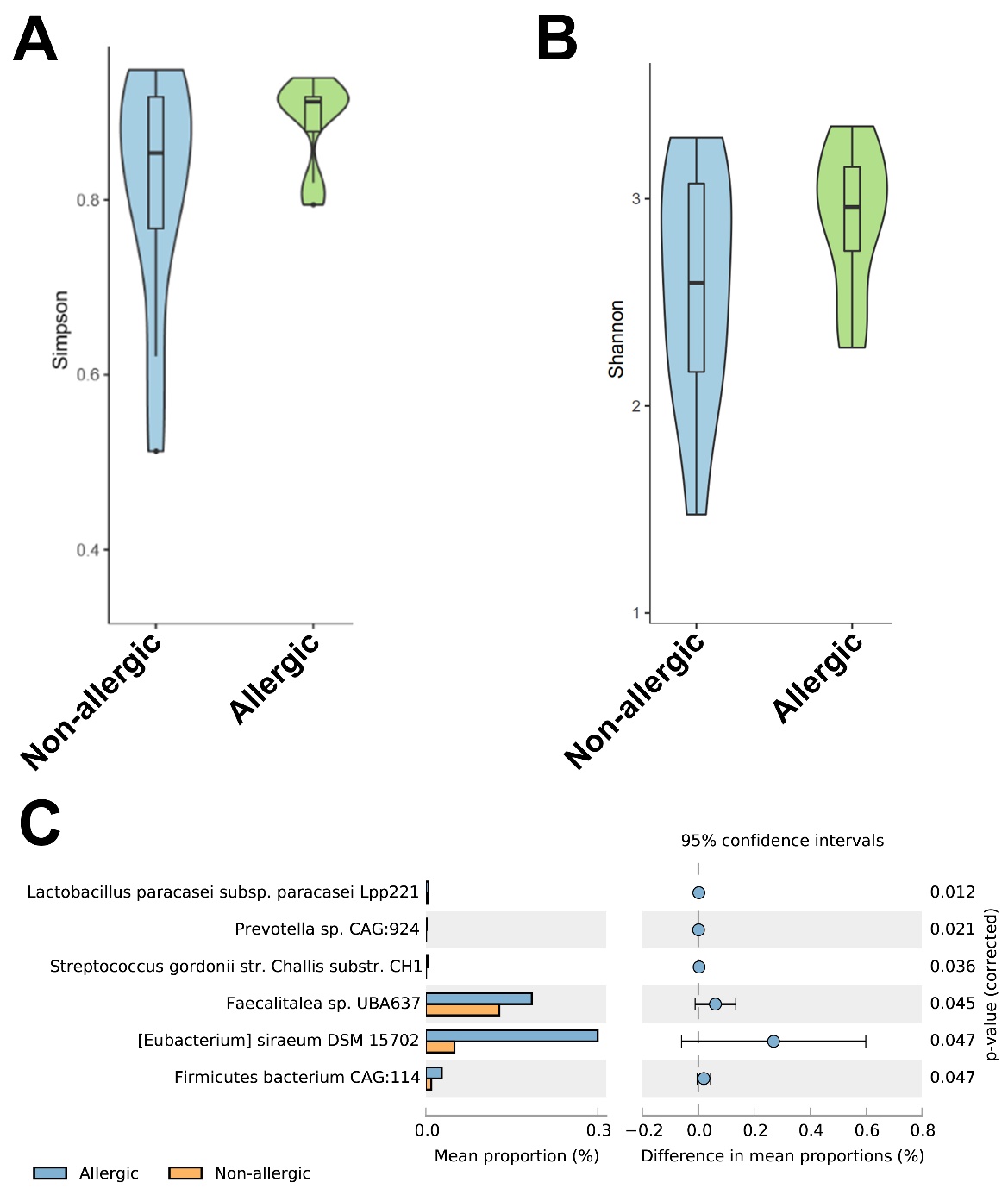


**Supplementary Figure 1. The microbiota composition in cohort study B. (A)** The Simpson index of the microbiota. **(B)** The Shannon index of the microbiota. **(C)** Differential species between non-allergic and allergic groups.
